# Cell therapy for neurological disorders: Progress towards an embryonic medial ganglionic eminence progenitor-based treatment

**DOI:** 10.3389/fnins.2023.1177678

**Published:** 2023-04-14

**Authors:** Joseane Righes Marafiga, Scott C. Baraban

**Affiliations:** ^1^Department of Neurological Surgery, University of California San Francisco, San Francisco, CA, United States; ^2^Helen Wills Institute for Neuroscience, University of California Berkeley, Berkeley, CA, United States

**Keywords:** interneuron, GABA, porcine, progenitor cells, epilepsy

## Abstract

Impairment of development, migration, or function of inhibitory interneurons are key features of numerous circuit-based neurological disorders, such as epilepsy. From a therapeutic perspective, symptomatic treatment of these disorders often relies upon drugs or deep brain stimulation approaches to provide a general enhancement of GABA-mediated inhibition. A more effective strategy to target these pathological circuits and potentially provide true disease-modifying therapy, would be to selectively add new inhibitory interneurons into these circuits. One such strategy, using embryonic medial ganglionic (MGE) progenitor cells as a source of a unique sub-population of interneurons, has already proven effective as a cell transplantation therapy in a variety of preclinical models of neurological disorders, especially in mouse models of acquired epilepsy. Here we will discuss the evolution of this interneuron-based transplantation therapy in acquired epilepsy models, with an emphasis on the recent adaptation of MGE progenitor cells for xenotransplantation into larger mammals.

## Introduction

Interneurons mediate inhibitory synaptic transmission, brain oscillations and cortical processing (Isaacson and Scanziani, 2011). Alteration of GABAergic transmission, following interneuron loss or dysfunction, can disrupt synaptic inhibition within neuronal networks and facilitate development of epilepsy (Kumar and Buckmaster, 2006; Knopp et al., 2008; Katsarou et al., 2017), autism spectrum disorders (Paterno et al., 2021; Fontes-Dutra et al., 2023), schizophrenia (Nakazawa et al., 2012) and other neurological conditions (Calcagnotto et al., 2005; Marín, 2012). Among these, epilepsy is a common and devastating condition, affecting more than 70 million people worldwide and approximately 3.4 million people in the United States (Ngugi et al., 2011; Zack and Kobau, 2017). As a first line treatment, antiseizure medications (ASMs) potentiating GABAergic inhibition are routinely used to control seizures (Löscher and Klein, 2021). However, alternative treatments are needed for drug-resistant epilepsy patients comprising nearly 30% of this patient population (Kwan and Brodie, 2000). In a select group of these patients, surgical removal of seizure foci is considered the best available treatment (Wiebe et al., 2001, Spencer and Huh, 2008, Jobst and Cascino, 2015). While a well-established procedure, invasive surgical resection carries significant risk for cognitive and psychological impact (Sherman et al., 2011); and its success is directly correlated with identification of seizure foci, which cannot always be defined or safely removed (Rosenow and Lüders, 2001). Deep brain stimulation (DBS) also provides a degree of seizure control in some patients by targeting propagation points within the epileptic network but is also invasive and not selective to inhibitory or excitatory pathways (Piper et al., 2022). In recent years, a promising alternative therapy using GABAergic progenitor cell-based transplantation has emerged with potential to selectively add new functional inhibitory neurons into the epileptic network (Baraban et al., 2009; Hunt et al., 2013; Casalia et al., 2017).

Preclinical transplantation studies using murine embryonic progenitors from medial ganglionic eminence (MGE) consistently demonstrate robust abilities to (i) migrate, differentiate and integrate into host circuits as inhibitory interneurons (Alvarez-Dolado et al., 2006; Calcagnotto et al., 2010), (ii) make synaptic inhibitory connections onto host brain excitatory neurons (Howard and Baraban, 2016; Hsieh and Baraban, 2017), (iii) enhance synaptic inhibition (Alvarez-Dolado et al., 2006; Calcagnotto et al., 2010; Howard et al., 2014) and (iv) abolish spontaneous seizures and epilepsy-related comorbidities in mouse models of acquired epilepsy (Baraban et al., 2009; Hunt et al., 2013; Casalia et al., 2017). Based on these investigations, transplanted MGE-derived interneurons are therapeutic because they increase surround inhibition in host brain by forming new inhibitory synapses within the epileptic network while other proposed mechanisms such as secretion of unidentified “trophic factors” or “reorganization of host circuitry” (Southwell et al., 2010, 2014; Priya et al., 2019) are not supported by experimental evidence. While these proof-of-principle studies involved embryonic donor cells from mice transplanted into recipient mice, moving to a larger mammal requires a cross-species xenotransplantation strategy. In our efforts to translate this promising therapy to larger mammals, we focused our attention on porcine donors as there is significant translational clinical precedent for using tissue or organs derived from pigs for xenotransplantation. In this mini-review, we discuss our earlier MGE transplantation work using mice and more recent studies highlighting the potential for porcine MGE xenotransplantation as a therapy for intractable epilepsies.

## Embryonic MGE progenitors transplantation therapy

During development, MGE is a primary source of inhibitory interneuron progenitors, generating primarily parvalbumin-expressing (PV) and somatostatin-expressing (SOM) cells (Wonders and Anderson, 2006). Over the past 20 years, studies utilizing embryonic murine MGE progenitors were extremely successful in demonstrating that MGE-derived cells are valuable candidates for interneuron transplantation therapies (Alvarez-Dolado et al., 2006; Martínez-Cerdeño et al., 2010; Zipancic et al., 2010; Bráz et al., 2012; Hunt et al., 2013; Hunt and Baraban, 2015; Casalia et al., 2017; Zhu et al., 2019; Paterno et al., 2020). Transplanted MGE progenitors offer widespread therapeutic coverage in a dysfunctional brain as embryonic MGE-derived progenitors survive (up to 22%) and migrate (up to 5 mm from injection site) extensively within cortical and hippocampal regions following transplantation (Alvarez-Dolado et al., 2006; Baraban et al., 2009; Hunt et al., 2013). In nearly two decades of investigation, embryonic MGE-derived cells have never been shown to proliferate in the host brain, but consistently share morphological features of native interneurons, with elaborated dendritic trees, and typical interneuron morphologies: bipolar cells, multipolar cells, basket cells, neurons with small body or chandelier cells (Alvarez-Dolado et al., 2006; Calcagnotto et al., 2010; Hunt et al., 2013). Most importantly, interneurons derived from embryonic murine MGE progenitors express the primary inhibitory neurotransmitter GABA and differentiate into a specific sub-population of PV-and SOM-positive GABAergic interneurons (Table 1). MGE-derived interneurons maintain electrophysiological properties of mature interneurons *in vitro*, such as fast-spiking and regular-spiking firing properties in current-clamp recordings (Alvarez-Dolado et al., 2006; Hunt et al., 2013; Howard and Baraban, 2016), and receive both inhibitory and excitatory synaptic input from host brain neurons as measured by voltage-clamp recordings of postsynaptic current (Martínez-Cerdeño et al., 2010; Sebe and Baraban, 2010; Howard and Baraban, 2016). Importantly, murine MGE-derived interneurons do not enhance GABA-mediated synaptic inhibition to endogenous interneurons which would suggest a caudal ganglionic eminence (CGE) interneuron lineage (Baraban et al., 2009; Larimer et al., 2016; Hsieh and Baraban, 2017). Enhancement of GABA-mediated inhibition following transplantation of MGE progenitors is also ‘self-limiting’ in that increased inhibition observed in host circuits does not scale with injection size but reaches a plateau (Sebe et al., 2014; Southwell et al., 2014). As expected for these interneuron sub-populations, MGE-derived interneurons integrate in hosts brain to mediate synaptic inhibition onto somatic and dendritic postsynaptic neuronal compartments of principal excitatory neurons as shown in dual patch-clamp recordings (Howard and Baraban, 2016) and optogenetic stimulation of transplant-derived interneurons (Hsieh and Baraban, 2017). Moreover, as a basis for therapeutic application in conditions where synaptic inhibition may be compromised (e.g., epilepsy, autism or schizophrenia), MGE-derived interneurons consistently enhance miniature and spontaneous inhibitory postsynaptic currents onto excitatory (but not inhibitory) pyramidal neurons in host brain (Alvarez-Dolado et al., 2006; Hunt et al., 2013; Howard and Baraban, 2016; Casalia et al., 2017; Hsieh and Baraban, 2017). The consistency of these findings across time and different investigators sets a standard for how any transplantation protocol purporting to utilize MGE progenitor cells should be judged.

**Table 1:** Medial ganglionic eminence.

Cell source	Author, year	Recipient, age	Interneuron markers	Additional properties
Murine embryonic progenitor cells	Alvarez-Dolado et al. (2006)	CD-1 mice, P3-4	CTX: GABA (68.6%) PV (38.3%); SOM (43.2%); CR (1.9%); NPY (7.8%). HP: GABA (42.8%); PV (33.7%); SOM (33.8%); CR (10.3%); NPY (13.1%).	Migration >5 mm from injection site
				Regular-spiking non-pyramidal, burst-onset regular-spiking non-pyramidal and fast-spiking firing properties
	Calcagnotto et al. (2010)	CD-1 mice, P60	HP: GABA (54.6%); PV (34.6%); SOM (34.3%); CR (3.5%); NPY (18.8%)	Fully mature morphology
	Hunt et al. (2013)	CD-1 mice, P60	HP: GAD67 (63%); PV (7.7%); SOM (41.3%); nNOS (21.6%); CR (6.4%); NPY (7.5%); Reelin (12.5%); VIP (0%).	Migration >1.5 mm from injection site
				Regular-spiking non-pyramidal, burst-spiking, late-spiking and fast-spiking firing properties
	Howard and Baraban (2016)	CD-1 mice, P1-3	HP: GAD67 (61.2%); PV (22.5%); SOM (36.2%); CR (6.98%); NPY (3.56%)	Regular-spiking non-pyramidal and fast-spiking firing properties
	Hsieh and Baraban (2017)	CD-1 mice, P2	HP: PV (28.8%); SOM (36.3%); nNOS (9.4%); CR (5.4%); Reelin (10.7%); VIP (0.25%).	Functional inhibitory connections with native pyramidal neurons
Human embryonic stem cells	Maroof et al. (2013)	NOD-SCID IL2RG^−/−^ mice, P2	CTX: GABA (90.2%) no PV or SOM cells	“Physiologically active neurons”
				Undifferentiated bipolar or unipolar morphologies
	Nicholas et al. (2013)	SCID mice, P2	CTX: GABA (50.9%); PV (not reported); SOM (50.1%); CB (60.9%); CR (72.5%).	“A fraction of human cells migrated more than 1 mm”
				Significant delay to spike at threshold and little adaptation (Type I) and rapid adaptation (Type II) firing properties

P: postnatal day; CTX: cortex; HP: hippocampus; GABA: gamma-aminobutyric acid; PV: parvalbumin; SOM: somatostatin; CR: calretinin; VIP: vasoactive intestinal peptide; NPY: neuropeptide Y; nNOS: neuronal nitric oxide synthase; CB: calbindin.

Mice provide unique advantages for development of MGE-derived transplantation protocols as a potential disease modifying therapy for epilepsy. First, our understanding of interneuron origins, sub-populations and functions derive from a wealth of rodent publications. Second, a variety of rodent epilepsy models are already available and easily accessible. As discussed, anatomical, immunohistochemical and functional properties of transplanted murine MGE-derived interneurons are now established (Alvarez-Dolado et al., 2006; Howard et al., 2014; Howard and Baraban, 2016; Hsieh and Baraban, 2017). As are studies (replicated over several publications) that transplanted murine MGE progenitors offer a potential cure in mouse models of epilepsy. One of the most studied rodent epilepsy models representing temporal lobe epilepsy (TLE), is the pilocarpine model (Turski et al., 1983; Cavalheiro et al., 1991; Cavalheiro, 1995; Sanabria et al., 2002). Using this model we demonstrated that at 60 days following transplantation, adult epileptic mice receiving MGE progenitor cells (after the emergence of documented epileptic phenotypes) exhibit a 92% reduction in spontaneous seizure frequency as measured by video-EEG monitoring and rescue of epilepsy-related comorbid behaviors in a variety of established assays including open field and Morris-Water maze deficits (Hunt et al., 2013). Despite some initial unfounded concerns following this report questioning “whether transplanted interneurons can integrate into neural circuits affected by long-standing epilepsy, or whether they exert a long-lasting effect on seizure phenotypes” (Henderson et al., 2014; Southwell et al., 2014), these effects were robust. Indeed in 2017, again using the pilocarpine model, we reported that embryonic MGE transplantation into adult epileptic mice produced an 84 to 88% reduction in spontaneous seizure frequency, again rescued open field and handling comorbid behaviors, and functionally restored GABA-mediated inhibition to levels similar to age-matched naïve controls. These effects, noted at 60 to 240 days after transplantation, demonstrate a clear functional restoration of synaptic inhibition in a hippocampal network affected by seizures and a persistent rescue of epilepsy phenotypes (Casalia et al., 2017).

Potential physiological incompatibility and ethical issues that come with murine progenitor cells limit the translation of this approach to a larger mammal. While some would argue that human induced pluripotent stem cells (hiPSC) could solve these issues, published hiPSC protocols do not convincingly report data for PV-and SOM-expressing neurons with the ability to migrate widely and functionally integrate as mature interneurons following transplantation. Despite Waldau et al. (2010) claims that the “majority” of hiPSC-derived cells become GABAergic cells, only 10% of these cells co-labeled with an antibody recognizing GABA while, inconsistent with an MGE lineage, 57% of these cells co-labeled with an antibody recognizing S-100B (Waldau et al., 2010). In Nicholas et al. (2013), following transplantation into immune-deficient mice only a very small fraction of hiPSC-derived putative “MGE-like” cells survive (3–9%), PV-positive neurons were not identified and 73% of cells expressed calretinin (e.g., an interneuron marker for cells originating in CGE) (Nicholas et al., 2013). Additionally, Maroof et al. (2013) demonstrated that hiPSC-derived neurons did not differentiate morphologically into interneurons, nor express SOM or PV (Maroof et al., 2013). Putative human iPSC-derived “MGE” cells in Upadhya et al. (2019) expanded by ~129% following intra-hippocampal grafting suggesting that these stem cells continued to proliferate in the host brain and could result in teratoma formation (Upadhya et al., 2019). Functional integration and enhancement of synaptic inhibition in host brain receiving these hiPSC-derived cells was not demonstrated in any of these studies. Finally, a recent single-nuclei and single-cell transcriptomic analysis of these published hiPSC-derived interneuron protocols (Allison et al., 2021) concluded that “none of these methods produced mature, postnatal-like interneurons as defined functionally (electrophysiology) or transcriptionally.” Therefore, current human iPSC protocols do not faithfully recapitulate crucial properties of embryonic MGE progenitors, and should be interpreted with a note of caution before being used as a strategy for interneuron-based transplant therapy in patients with epilepsy, and other neurological disorders, such as ASD, Alzheimer’s disease and schizophrenia.

## Porcine MGE progenitor cells

Porcine xenotransplantation studies raise the possibility of developing safe and effective human therapies for several neurological disorders (Fink et al., 2000; Cooper et al., 2002; Hryhorowicz et al., 2017; Sykes and Sachs, 2019; Casalia et al., 2021; Simeone et al., 2022). As such, our laboratory recently turned our attention to using porcine embryonic tissue as an MGE progenitor source for xenotransplantation. In Casalia et al. (2021), we demonstrated that porcine MGE-derived cells recapitulate molecular, morphological and immunohistochemical profiles similar to those established for murine MGE-derived cells (Casalia et al., 2021). First, embryonic porcine MGE express transcription factors, such as *Nkx2.1*, *Lhx6*, and Dlx2, indicative of an MGE lineage (Cobos et al., 2005, 2006, 2007; Vogt et al., 2014). Second, *in vitro* and *in vivo* protocols show that porcine MGE progenitors exhibit a robust migratory capacity similar to murine MGE progenitors (Wichterle et al., 1999, 2001; Alvarez-Dolado et al., 2006; Hunt and Baraban, 2015). Third, under appropriate *in vitro* induction protocols, porcine MGE progenitors differentiate into cortical or hippocampal GABAergic with mature interneuron-like morphologies and expression of GABA, SOM and the vesicular GABA transporter (vGAT) (Casalia et al., 2021). Fourth, following xenotransplantation into adult rat hippocampus, porcine MGE-derived neurons migrate, display morphological similarities similar to mature native interneurons (e.g., basket cells, multipolar neurons, bipolar cells) and express both GABA and SOM (Alvarez-Dolado et al., 2006; Hunt et al., 2013; Casalia et al., 2021). Note, commercially available antibodies recognizing porcine PV-expressing neurons were not selective enough to evaluate this sub-population in these studies. Further, porcine MGE-derived neurons did not exhibit morphologic features of mature cortical pyramidal neurons and tumors were not observed in any host animal receiving porcine MGE progenitors. Together, these findings confirm that MGE-derived interneuron development is conserved across species. Future studies should evaluate the intrinsic electrophysiological properties and functional enhancement of synaptic inhibition for these porcine MGE-derived interneurons following xenotransplantation.

While additional characterization of porcine MGE progenitors awaits, a highly successful N-of-1 trial was recently reported for a refractory, domoic-acid poisoned epileptic California sea-lion playfully named Cronutt (Simeone et al., 2022). Domoic-acid poisoned sea lions consistently present with spontaneous convulsive seizures, hippocampal atrophy including loss of hippocampal PV-and SOM-expressing interneurons, and cognitive/behavioral deficits, similar to patients with TLE (de Lanerolle et al., 2003; Buckmaster et al., 2014; Cendes et al., 2014; Ives-Deliperi and Butler, 2021). Cronutt, rescued initially by the Marine Mammal Center and eventually adopted by Six Flags Discovery Kingdome presented with uncontrollable convulsive seizures (up to 10 per day), hippocampal atrophy (Cook et al., 2021), lethargy and severe weight loss. After considering euthanasia, we developed a protocol for stereotaxic-guided xenotransplantation of embryonic porcine MGE progenitors in a sea lion. In the initial case report at 12 months following xenotransplantation, handlers at the Six Flags pinniped facility reported Cronutt’s complete absence of convulsive seizures, with significant improvements in appetite, weight gain, and general behaviors (Simeone et al., 2022). Anecdotal observations now stretching beyond 28 months post xenotransplantation continue to report a seizure-free outcome and significant improvements in behavior and cognition.

## Conclusion

**Figure 1:**
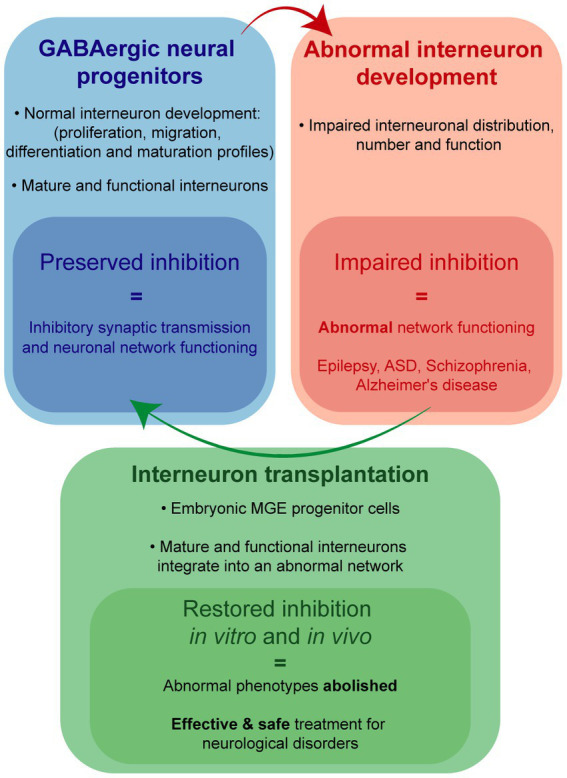
Interneuron-based cell transplantation therapy.

While precise mechanism(s) by which interneuron dysfunction contributes to epilepsy (and related neurological disorders) remains an active area of investigation, an aspirational treatment goal is a “no seizures, no side effects” outcome. Although this goal remains elusive, patient and animal model data converge on a hypothesis that such a treatment could involve enhancement of GABA-mediated synaptic inhibition. Transplanted embryonic MGE progenitors have consistently been shown to migrate and functionally integrate in host circuits to enhance synaptic inhibition in a manner resembling that possible with native interneurons. Properties of interneurons derived from murine and porcine MGE progenitors make them ideal candidates for a cell transplantation treatment (Figure 1). Indeed, MGE transplantation drastically reduced seizures, abolished epilepsy-related comorbidities and failed to show any adverse side effects in experimental rodent models of acquired epilepsy and the case of a naturally occurring large mammal with TLE. Therefore, we are optimistic embryonic MGE progenitors offer a safe and effective means toward developing a disease-modifying (no seizures, no side effects) treatment for intractable epilepsies.

## Author contributions

JR wrote the first draft of the manuscript. SB wrote and reviewed the final version of the manuscript. All authors contributed to the article and approved the submitted version.

## Funding

This work was supported by NIH/NINDS grant R01 NS-071785–13 to SB.

## Conflict of interest

The authors declare that the research was conducted in the absence of any commercial or financial relationships that could be construed as a potential conflict of interest.

## Publisher’s note

All claims expressed in this article are solely those of the authors and do not necessarily represent those of their affiliated organizations, or those of the publisher, the editors and the reviewers. Any product that may be evaluated in this article, or claim that may be made by its manufacturer, is not guaranteed or endorsed by the publisher.

## References

[ref1] Allison T. Langerman J. Sabri S. Otero-Garcia M. Lund A. Huang J.. (2021). Defining the nature of human pluripotent stem cell-derived interneurons via single-cell analysis. Stem Cell Rep. 16, 2548–2564. doi: 10.1016/j.stemcr.2021.08.006, PMID: 34506726 PMC8514853

[ref2] Alvarez-Dolado M. Calcagnotto M. E. Karkar K. M. Southwell D. G. Jones-Davis D. M. Estrada R. C.. (2006). Cortical inhibition modified by embryonic neural precursors grafted into the postnatal brain. J. Neurosci. 26, 7380–7389. doi: 10.1523/JNEUROSCI.1540-06.2006, PMID: 16837585 PMC1550786

[ref3] Baraban S. C. Southwell D. G. Estrada R. C. Jones D. L. Sebe J. Y. Alfaro-Cervello C.. (2009). Reduction of seizures by transplantation of cortical GABAergic interneuron precursors into Kv1.1 mutant mice. Proc. Natl. Acad. Sci. U. S. A. 106, 15472–15477. doi: 10.1073/pnas.0900141106, PMID: 19706400 PMC2741275

[ref4] Bráz J. M. Sharif-Naeini R. Vogt D. Kriegstein A. Alvarez-Buylla A. Rubenstein J. L.. (2012). Forebrain GABAergic neuron precursors integrate into adult spinal cord and reduce injury-induced neuropathic pain. Neuron 74, 663–675. doi: 10.1016/j.neuron.2012.02.033, PMID: 22632725 PMC3361692

[ref5] Buckmaster P. S. Wen X. Toyoda I. Gulland F. M. van Bonn W. (2014). Hippocampal neuropathology of domoic acid-induced epilepsy in California Sea lions (*Zalophus californianus*). J. Comp. Neurol. 522, 1691–1706. doi: 10.1002/cne.23509, PMID: 24638960 PMC4100486

[ref6] Calcagnotto M. E. Paredes M. F. Tihan T. Barbaro N. M. Baraban S. C. (2005). Dysfunction of synaptic inhibition in epilepsy associated with focal cortical dysplasia. J. Neurosci. 25, 9649–9657. doi: 10.1523/JNEUROSCI.2687-05.2005, PMID: 16237169 PMC6725719

[ref7] Calcagnotto M. E. Zipancic I. Piquer-Gil M. Mello L. E. Alvarez-Dolado M. (2010). Grafting of GABAergic precursors rescues deficits in hippocampal inhibition. Epilepsia 51, 66–70. doi: 10.1111/j.1528-1167.2010.02613.x, PMID: 20618404

[ref8] Casalia M. L. Howard M. A. Baraban S. C. (2017). Persistent seizure control in epileptic mice transplanted with gamma-aminobutyric acid progenitors. Ann. Neurol. 82, 530–542. doi: 10.1002/ana.25021, PMID: 28833459 PMC5771437

[ref9] Casalia M. L. Li T. Ramsay H. Ross P. J. Paredes M. F. Baraban S. C. (2021). Interneuron origins in the embryonic porcine medial ganglionic Eminence. J. Neurosci. 41, 3105–3119. doi: 10.1523/JNEUROSCI.2738-20.2021, PMID: 33637558 PMC8026360

[ref10] Cavalheiro E. A. (1995). The pilocarpine model of epilepsy. Ital. J. Neurol. Sci. 16, 33–37. doi: 10.1007/BF02229072 7642349

[ref11] Cavalheiro E. A. Leite J. P. Bortolotto Z. A. Turski W. A. Ikonomidou C. Turski L. (1991). Long-term effects of pilocarpine in rats: structural damage of the brain triggers kindling and spontaneous recurrent seizures. Epilepsia 32, 778–782. doi: 10.1111/j.1528-1157.1991.tb05533.x, PMID: 1743148

[ref12] Cendes F. Sakamoto A. C. Spreafico R. Bingaman W. Becker A. J. (2014). Epilepsies associated with hippocampal sclerosis. Acta Neuropathol. 128, 21–37. doi: 10.1007/s00401-014-1292-0 24823761

[ref13] Cobos I. Borello U. Rubenstein J. L. (2007). Dlx transcription factors promote migration through repression of axon and dendrite growth. Neuron 54, 873–888. doi: 10.1016/j.neuron.2007.05.024, PMID: 17582329 PMC4921237

[ref14] Cobos I. Calcagnotto M. E. Vilaythong A. J. Thwin M. T. Noebels J. L. Baraban S. C.. (2005). Mice lacking Dlx1 show subtype-specific loss of interneurons, reduced inhibition and epilepsy. Nat. Neurosci. 8, 1059–1068. doi: 10.1038/nn1499, PMID: 16007083

[ref15] Cobos I. Long J. E. Thwin M. T. Rubenstein J. L. (2006). Cellular patterns of transcription factor expression in developing cortical interneurons. Cereb. Cortex 16, i82–i88. doi: 10.1093/cercor/bhk003 16766712

[ref16] Cook P. F. Hoard V. A. Dolui S. Frederick B. D. Redfern R. Dennison S. E.. (2021). An MRI protocol for anatomical and functional evaluation of the California Sea lion brain. J. Neurosci. Methods 353:109097. doi: 10.1016/j.jneumeth.2021.109097, PMID: 33581216

[ref17] Cooper D. K. Gollackner B. Knosalla C. Teranishi K. (2002). Xenotransplantation--how far have we come? Transpl. Immunol. 9, 251–256. doi: 10.1016/S0966-3274(02)00010-2 12180839

[ref18] De Lanerolle N. C. Kim J. H. Williamson A. Spencer S. S. Zaveri H. P. Eid T.. (2003). A retrospective analysis of hippocampal pathology in human temporal lobe epilepsy: evidence for distinctive patient subcategories. Epilepsia 44, 677–687. doi: 10.1046/j.1528-1157.2003.32701.x 12752467

[ref19] Fink J. S. Schumacher J. M. Ellias S. L. Palmer E. P. Saint-Hilaire M. Shannon K.. (2000). Porcine xenografts in Parkinson's disease and Huntington's disease patients: preliminary results. Cell Transplant. 9, 273–278. doi: 10.1177/096368970000900212, PMID: 10811399

[ref20] Fontes-Dutra M. Righes Marafiga J. Santos-Terra J. Deckmann I. Brum Schwingel G. Rabelo B.. (2023). GABAergic synaptic transmission and cortical oscillation patterns in the primary somatosensory area of a valproic acid rat model of autism spectrum disorder. Eur. J. Neurosci. 57, 527–546. doi: 10.1111/ejn.15893, PMID: 36504470

[ref21] Henderson K. W. Gupta J. Tagliatela S. Litvina E. Zheng X. Van Zandt M. A.. (2014). Long-term seizure suppression and optogenetic analyses of synaptic connectivity in epileptic mice with hippocampal grafts of GABAergic interneurons. J. Neurosci. 34, 13492–13504. doi: 10.1523/JNEUROSCI.0005-14.2014, PMID: 25274826 PMC4180479

[ref22] Howard M. A. Baraban S. C. (2016). Synaptic integration of transplanted interneuron progenitor cells into native cortical networks. J. Neurophysiol. 116, 472–478. doi: 10.1152/jn.00321.2016, PMID: 27226453 PMC4978788

[ref23] Howard M. A. Rubenstein J. L. Baraban S. C. (2014). Bidirectional homeostatic plasticity induced by interneuron cell death and transplantation *in vivo*. Proc. Natl. Acad. Sci. U. S. A. 111, 492–497. doi: 10.1073/pnas.1307784111, PMID: 24344303 PMC3890856

[ref24] Hryhorowicz M. Zeyland J. Słomski R. Lipiński D. (2017). Genetically modified pigs as organ donors for xenotransplantation. Mol. Biotechnol. 59, 435–444. doi: 10.1007/s12033-017-0024-9, PMID: 28698981 PMC5617878

[ref25] Hsieh J. Y. Baraban S. C. (2017). Medial ganglionic Eminence progenitors transplanted into hippocampus integrate in a functional and subtype-appropriate manner. eNeuro:4:359. doi: 10.1523/ENEURO.0359-16.2017 PMC5388838 28413826

[ref26] Hunt R. F. Baraban S. C. (2015). Interneuron transplantation as a treatment for epilepsy. Cold Spring Harb. Perspect. Med.:a022376:5. doi: 10.1101/cshperspect.a022376 26627452 PMC4665034

[ref27] Hunt R. F. Girskis K. M. Rubenstein J. L. Alvarez-Buylla A. Baraban S. C. (2013). GABA progenitors grafted into the adult epileptic brain control seizures and abnormal behavior. Nat. Neurosci. 16, 692–697. doi: 10.1038/nn.3392, PMID: 23644485 PMC3665733

[ref28] Isaacson J. S. Scanziani M. (2011). How inhibition shapes cortical activity. Neuron 72, 231–243. doi: 10.1016/j.neuron.2011.09.027, PMID: 22017986 PMC3236361

[ref29] Ives-Deliperi V. Butler J. T. (2021). Mechanisms of cognitive impairment in temporal lobe epilepsy: a systematic review of resting-state functional connectivity studies. Epilepsy Behav. 115:107686. doi: 10.1016/j.yebeh.2020.107686, PMID: 33360743

[ref30] Jobst B. C. Cascino G. D. (2015). Resective epilepsy surgery for drug-resistant focal epilepsy: a review. JAMA 313, 285–293. doi: 10.1001/jama.2014.17426 25602999

[ref31] Katsarou A. M. Moshé S. L. Galanopoulou A. S. (2017). Interneuronopathies and their role in early life epilepsies and neurodevelopmental disorders. Epilepsia Open 2, 284–306. doi: 10.1002/epi4.12062, PMID: 29062978 PMC5650248

[ref32] Knopp A. Frahm C. Fidzinski P. Witte O. W. Behr J. (2008). Loss of GABAergic neurons in the subiculum and its functional implications in temporal lobe epilepsy. Brain 131, 1516–1527. doi: 10.1093/brain/awn095, PMID: 18504292

[ref33] Kumar S. S. Buckmaster P. S. (2006). Hyperexcitability, interneurons, and loss of GABAergic synapses in entorhinal cortex in a model of temporal lobe epilepsy. J. Neurosci. 26, 4613–4623. doi: 10.1523/JNEUROSCI.0064-06.2006, PMID: 16641241 PMC6674073

[ref34] Kwan P. Brodie M. J. (2000). Early identification of refractory epilepsy. N. Engl. J. Med. 342, 314–319. doi: 10.1056/NEJM200002033420503 10660394

[ref35] Larimer P. Spatazza J. Espinosa J. S. Tang Y. Kaneko M. Hasenstaub A. R.. (2016). Caudal ganglionic Eminence precursor transplants disperse and integrate as lineage-specific interneurons but do not induce cortical plasticity. Cell Rep. 16, 1391–1404. doi: 10.1016/j.celrep.2016.06.071, PMID: 27425623 PMC5047519

[ref36] Löscher W. Klein P. (2021). The pharmacology and clinical efficacy of Antiseizure medications: from bromide salts to Cenobamate and beyond. CNS Drugs 35, 935–963. doi: 10.1007/s40263-021-00827-8, PMID: 34145528 PMC8408078

[ref37] Marín O. (2012). Interneuron dysfunction in psychiatric disorders. Nat. Rev. Neurosci. 13, 107–120. doi: 10.1038/nrn3155 22251963

[ref38] Maroof A. M. Keros S. Tyson J. A. Ying S. W. Ganat Y. M. Merkle F. T.. (2013). Directed differentiation and functional maturation of cortical interneurons from human embryonic stem cells. Cell Stem Cell 12, 559–572. doi: 10.1016/j.stem.2013.04.008, PMID: 23642365 PMC3681523

[ref39] Martínez-Cerdeño V. Noctor S. C. Espinosa A. Ariza J. Parker P. Orasji S.. (2010). Embryonic MGE precursor cells grafted into adult rat striatum integrate and ameliorate motor symptoms in 6-OHDA-lesioned rats. Cell Stem Cell 6, 238–250. doi: 10.1016/j.stem.2010.01.004, PMID: 20207227 PMC4075336

[ref40] Nakazawa K. Zsiros V. Jiang Z. Nakao K. Kolata S. Zhang S.. (2012). GABAergic interneuron origin of schizophrenia pathophysiology. Neuropharmacology 62, 1574–1583. doi: 10.1016/j.neuropharm.2011.01.022, PMID: 21277876 PMC3090452

[ref41] Ngugi A. K. Kariuki S. M. Bottomley C. Kleinschmidt I. Sander J. W. Newton C. R. (2011). Incidence of epilepsy: a systematic review and meta-analysis. Neurology 77, 1005–1012. doi: 10.1212/WNL.0b013e31822cfc90, PMID: 21893672 PMC3171955

[ref42] Nicholas C. R. Chen J. Tang Y. Southwell D. G. Chalmers N. Vogt D.. (2013). Functional maturation of hPSC-derived forebrain interneurons requires an extended timeline and mimics human neural development. Cell Stem Cell 12, 573–586. doi: 10.1016/j.stem.2013.04.005, PMID: 23642366 PMC3699205

[ref43] Paterno R. Casalia M. Baraban S. C. (2020). Interneuron deficits in neurodevelopmental disorders: implications for disease pathology and interneuron-based therapies. Eur. J. Paediatr. Neurol. 24, 81–88. doi: 10.1016/j.ejpn.2019.12.015, PMID: 31870698 PMC7152321

[ref44] Paterno R. Marafiga J. R. Ramsay H. Li T. Salvati K. A. Baraban S. C. (2021). Hippocampal gamma and sharp-wave ripple oscillations are altered in a Cntnap2 mouse model of autism spectrum disorder. Cell Rep. 37:109970. doi: 10.1016/j.celrep.2021.109970, PMID: 34758298 PMC8783641

[ref45] Piper R. J. Richardson R. M. Worrell G. Carmichael D. W. Baldeweg T. Litt B.. (2022). Towards network-guided neuromodulation for epilepsy. Brain 145, 3347–3362. doi: 10.1093/brain/awac234, PMID: 35771657 PMC9586548

[ref46] Priya R. Rakela B. Kaneko M. Spatazza J. Larimer P. Hoseini M. S.. (2019). Vesicular GABA transporter is necessary for transplant-induced critical period plasticity in mouse visual cortex. J. Neurosci. 39, 2635–2648. doi: 10.1523/JNEUROSCI.1253-18.2019, PMID: 30705101 PMC6445995

[ref47] Rosenow F. Lüders H. (2001). Presurgical evaluation of epilepsy. Brain 124, 1683–1700. doi: 10.1093/brain/124.9.1683 11522572

[ref48] Sanabria E. R. da Silva A. V. Spreafico R. Cavalheiro E. A. (2002). Damage, reorganization, and abnormal neocortical hyperexcitability in the pilocarpine model of temporal lobe epilepsy. Epilepsia 43, 96–106. doi: 10.1046/j.1528-1157.43.s.5.31.x 12121302

[ref49] Sebe J. Y. Baraban S. C. (2010). The promise of an interneuron-based cell therapy for epilepsy. Dev. Neurobiol. 71, 107–117. doi: 10.1002/dneu.20813 PMC3059084 21154914

[ref50] Sebe J. Y. Looke-Stewart E. Baraban S. C. (2014). GABAB receptors in maintenance of neocortical circuit function. Exp. Neurol. 261, 163–170. doi: 10.1016/j.expneurol.2014.05.018, PMID: 24873729 PMC4324605

[ref51] Sherman E. M. Wiebe S. Fay-Mcclymont T. B. Tellez-Zenteno J. Metcalfe A. Hernandez-Ronquillo L.. (2011). Neuropsychological outcomes after epilepsy surgery: systematic review and pooled estimates. Epilepsia 52, 857–869. doi: 10.1111/j.1528-1167.2011.03022.x, PMID: 21426331

[ref52] Simeone C. A. Andrews J. P. Johnson S. P. Casalia M. Kochanski R. Chang E. F.. (2022). Xenotransplantation of porcine progenitor cells in an epileptic California Sea lion (Zalophus californianus): illustrative case. J Neurosurg Case Lessons 3:1474. doi: 10.3171/CASE21417, PMID: 36273868 PMC9379678

[ref53] Southwell D. G. Froemke R. C. Alvarez-Buylla A. Stryker M. P. Gandhi S. P. (2010). Cortical plasticity induced by inhibitory neuron transplantation. Science 327, 1145–1148. doi: 10.1126/science.1183962, PMID: 20185728 PMC3164148

[ref54] Southwell D. G. Nicholas C. R. Basbaum A. I. Stryker M. P. Kriegstein A. R. Rubenstein J. L.. (2014). Interneurons from embryonic development to cell-based therapy. Science 344:1240622. doi: 10.1126/science.1240622, PMID: 24723614 PMC4056344

[ref55] Spencer S. Huh L. (2008). Outcomes of epilepsy surgery in adults and children. Lancet Neurol. 7, 525–537. doi: 10.1016/S1474-4422(08)70109-1 18485316

[ref56] Sykes M. Sachs D. H. (2019). Transplanting organs from pigs to humans. Sci. Immunol. 4:6298. doi: 10.1126/sciimmunol.aau6298, PMID: 31676497 PMC7293579

[ref57] Turski W. A. Cavalheiro E. A. Schwarz M. Czuczwar S. J. Kleinrok Z. Turski L. T. (1983). Limbic seizures produced by pilocarpine in rats: behavioural, electroencephalographic and neuropathological study. Behav. Brain Res. 9, 315–335. doi: 10.1016/0166-4328(83)90136-5, PMID: 6639740

[ref58] Upadhya D. Hattiangady B. Castro O. W. Shuai B. Kodali M. Attaluri S.. (2019). Human induced pluripotent stem cell-derived MGE cell grafting after status epilepticus attenuates chronic epilepsy and comorbidities via synaptic integration. Proc. Natl. Acad. Sci. U. S. A. 116, 287–296. doi: 10.1073/pnas.1814185115, PMID: 30559206 PMC6320542

[ref59] Vogt D. Hunt R. F. Mandal S. Sandberg M. Silberberg S. N. Nagasawa T.. (2014). Lhx6 directly regulates Arx and CXCR7 to determine cortical interneuron fate and laminar position. Neuron 82, 350–364. doi: 10.1016/j.neuron.2014.02.030, PMID: 24742460 PMC4261952

[ref60] Waldau B. Hattiangady B. Kuruba R. Shetty A. K. (2010). Medial ganglionic eminence-derived neural stem cell grafts ease spontaneous seizures and restore GDNF expression in a rat model of chronic temporal lobe epilepsy. Stem Cells 28, 1153–1164. doi: 10.1002/stem.446, PMID: 20506409 PMC2933789

[ref61] Wichterle H. Garcia-Verdugo J. M. Herrera D. G. Alvarez-Buylla A. (1999). Young neurons from medial ganglionic eminence disperse in adult and embryonic brain. Nat. Neurosci. 2, 461–466. doi: 10.1038/8131, PMID: 10321251

[ref62] Wichterle H. Turnbull D. H. Nery S. Fishell G. Alvarez-Buylla A. (2001). In utero fate mapping reveals distinct migratory pathways and fates of neurons born in the mammalian basal forebrain. Development 128, 3759–3771. doi: 10.1242/dev.128.19.3759, PMID: 11585802

[ref63] Wiebe S. Blume W. T. Girvin J. P. Eliasziw M. Effectiveness and Efficiency of Surgery for Temporal Lobe Epilepsy Study Group (2001). A randomized, controlled trial of surgery for temporal-lobe epilepsy. N. Engl. J. Med. 345, 311–318. doi: 10.1056/NEJM200108023450501 11484687

[ref64] Wonders C. P. Anderson S. A. (2006). The origin and specification of cortical interneurons. Nat. Rev. Neurosci. 7, 687–696. doi: 10.1038/nrn1954 16883309

[ref65] Zack M. M. Kobau R. (2017). National and state estimates of the numbers of adults and children with active epilepsy–United States, 2015. MMWR Morb. Mortal. Wkly Rep. 66, 821–825. doi: 10.15585/mmwr.mm6631a1, PMID: 28796763 PMC5687788

[ref66] Zhu B. Eom J. Hunt R. F. (2019). Transplanted interneurons improve memory precision after traumatic brain injury. Nat. Commun. 10:5156. doi: 10.1038/s41467-019-13170-w, PMID: 31727894 PMC6856380

[ref67] Zipancic I. Calcagnotto M. E. Piquer-Gil M. Mello L. E. Alvarez-Dolado M. (2010). Transplant of GABAergic precursors restores hippocampal inhibitory function in a mouse model of seizure susceptibility. Cell Transplant. 19, 549–564. doi: 10.3727/096368910X491383, PMID: 20144261

